# Porous Hollow Superlattice NiMn_2_O_4_/NiCo_2_O_4_ Mesocrystals as a Highly Reversible Anode Material for Lithium-Ion Batteries

**DOI:** 10.3389/fchem.2018.00153

**Published:** 2018-05-15

**Authors:** Lingjun Li, Qi Yao, Jiequn Liu, Kaibo Ye, Boyu Liu, Zengsheng Liu, Huiping Yang, Zhaoyong Chen, Junfei Duan, Bao Zhang

**Affiliations:** ^1^School of Materials Science and Engineering, Changsha University of Science and Technology, Changsha, China; ^2^Hunan Provincial Key Laboratory of Efficient and Clean Energy Utilization, Changsha University of Science and Technology, Changsha, China; ^3^School of Iron and Steel, Soochow University, Suzhou, China; ^4^School of Metallurgy and Environment, Central South University, Changsha, China

**Keywords:** lithium-ion battery, transition metal oxide, superlattice structure, hollow multi-porous architecture, electrochemical kinetics

## Abstract

As a promising high-capacity anode material for Li-ion batteries, NiMn_2_O_4_ always suffers from the poor intrinsic conductivity and the architectural collapse originating from the volume expansion during cycle. Herein, a combined structure and architecture modulation is proposed to tackle concurrently the two handicaps, via a facile and well-controlled solvothermal approach to synthesize NiMn_2_O_4_/NiCo_2_O_4_ mesocrystals with superlattice structure and hollow multi-porous architecture. It is demonstrated that the obtained NiCo_1.5_Mn_0.5_O_4_ sample is made up of a new mixed-phase NiMn_2_O_4_/NiCo_2_O_4_ compound system, with a high charge capacity of 532.2 mAh g^−1^ with 90.4% capacity retention after 100 cycles at a current density of 1 A g^−1^. The enhanced electrochemical performance can be attributed to the synergistic effects of the superlattice structure and the hollow multi-porous architecture of the NiMn_2_O_4_/NiCo_2_O_4_ compound. The superlattice structure can improve ionic conductivity to enhance charge transport kinetics of the bulk material, while the hollow multi-porous architecture can provide enough void spaces to alleviate the architectural change during cycling, and shorten the lithium ions diffusion and electron-transportation distances.

## Introduction

Nowadays, Graphite-based materials are the main anode material for current commercial lithium-ion battery (LIB) (Kang et al., 2006; Scrosati et al., 2011; Xie et al., 2012; Goodenough and Park, 2013; Yuan et al., 2014; Yan et al., 2017). However, the low theoretical specific capacity of 372 mAh g^−1^ and poor rate capability limit its application in next generation high energy density LIB (Mai et al., 2011; Pan et al., 2014; Liu et al., 2015; Ma Z. et al., 2015; Wang J. X. et al., 2016; Wu et al., 2017; Su et al., 2018). In this regard, a variety of simple metal oxides, such as tin oxide, manganese oxide, iron oxide and cobalt oxide with high energy density, have drawn much attention as the desired candidates for anode materials to satisfy the high energy storage requirements (Mai et al., 2011; Xie et al., 2012; Li Q. et al., 2013; Pan et al., 2014; Yuan et al., 2014; Liu et al., 2015; Ma Z. et al., 2015; Wang J. X. et al., 2016; Yan et al., 2017; Su et al., 2018). In addition, complex oxides, such as NiMn_2_O_4_, ZnCo_2_O_4_, CoMn_2_O_4_, ZnFe_2_O_4_, CuCo_2_O_4_, and ZnMn_2_O_4_, have shown better electronic conductivity and higher reversible capacity than those of single-component metal oxides, which could be ascribed to the multiple aliovalent cations and corresponding more versatile redox reactions (Zhou et al., 2012; Li J. F. et al., 2013a,b,c; Chen et al., 2014c; Shen et al., 2014, 2015; Yuan et al., 2014; Zhang et al., 2014, 2015, 2016; Kang et al., 2015; Leng et al., 2015; Ma Y. et al., 2015; Ma Z. et al., 2015; Wang Y. K. et al., 2016; Wu L. J. et al., 2016; Yan et al., 2017). Among them, as a typical spinel structure, NiMn_2_O_4_ has been extensively studied and demonstrated as a promising next generation anode material for LIBs (Kang et al., 2015; Ma Z. et al., 2015; Shen et al., 2015). However, the poor intrinsic electronic conductivity of NiMn_2_O_4_ still impairs the electron transport kinetics during the process of the redox reaction, and NiMn_2_O_4_ shows rapid capacity fading because of the slow ion-diffusion rates and large volume changes during the cycling process (Kang et al., 2015; Ma Y. et al., 2015; Ma Z. et al., 2015; Shen et al., 2015; Zhang et al., 2015).

To solve the aforementioned problems and enhance the electrochemical performance of transition metal oxides, one efficient method is to synthesize hollow multi-porous architecture of transition metal oxides (Zhou et al., 2012; Choi and Kang, 2013; Li J. F. et al., 2013a,b,c; Li Q. et al., 2013; Zhu et al., 2013a,b; Chen et al., 2014c; Luo S. et al., 2014; Pan et al., 2014; Wang L. Y. et al., 2014; Zhang et al., 2014, 2015, 2018; Gao et al., 2015; Kang et al., 2015; Leng et al., 2015; Ma Z. et al., 2015; Shen et al., 2015; Su et al., 2015; Wang Y. K. et al., 2016; Wu et al., 2018). Compared with bulk counterparts, the hollow multi-porous structures can provide enough void space to buffer architectural changes and alleviate the formation of the aggregate particles during the process of redox reactions (Li J. F. et al., 2013c; Li Q. et al., 2013; Zhang et al., 2014, 2015, 2018; Kang et al., 2015; Leng et al., 2015; Ma Z. et al., 2015; Shen et al., 2015; Wang Y. K. et al., 2016). In addition, the hollow multi-porous character is conducive to the electrolyte penetration, offering a short path for the lithium ions diffusion and electron-transportation (Li J. F. et al., 2013c; Li Q. et al., 2013; Gao et al., 2015; Zhang et al., 2015, 2018; Wang Y. K. et al., 2016).

Most recently, cation substitution has been certified to be an effective route to improve the electrical conductivity and charge transfer ability of the anode materials (Mai et al., 2011; Qiu et al., 2011; Li et al., 2012, 2015, 2016; Li Q. et al., 2013; Reddy et al., 2013; Suo et al., 2013; Chen et al., 2014a,b; Liu et al., 2014; Sun et al., 2014; Wang J. X. et al., 2014; Xu et al., 2014; Choi et al., 2015; He et al., 2015, 2017; Ma Z. et al., 2015; Mo et al., 2015; Jin et al., 2016; Wu F. X. et al., 2016; Wu L. J. et al., 2016). Ma Y. et al. (2015) found that the optimal level of iron doping in spinel NiMn_2_O_4_ can improve the electrical conductivity and structural robustness and exhibit exciting lifespan. Tu et al. (Mai et al., 2011) found that “Co-doped NiO nanoflake arrays samples showed excellent rate capability and good capacity retention.” Furthermore, it is noted that the content of Co could also affect the structure and morphology of the transition metal oxides. Qian et al. (Li J. F. et al., 2013b) found that the distribution and morphology of MnCo_2_O_4_ particles are very different from those of CoMn_2_O_4_ particles. Typically, “the resultant pores are much larger in CoMn_2_O_4_ than that of MnCo_2_O_4_, possibly due to the longer transfer distance of ions” (Li J. F. et al., 2013b). These results imply that Co substitution is critical to the electrical conductivity and controlled porous architecture of transition metal oxides. However, to the best of our knowledge, the studies on Co substituted NiMn_2_O_4_ mesocrystals with controlled phase structure and morphology are scarcely reported.

Herein, we first report a solvothermal method for the successful preparation of well-distributed hollow multi-porous superlattice NiMn_2_O_4_/NiCo_2_O_4_ mesocrystals. As expected, Co element is uniformly distributed among the NiMn_2_O_4_ mesocrystals, and that Co substitution induces a great evolution of the structure and morphology. The structure is changed from the single-phase NiMn_2_O_4_ to a new mixed-phase NiMn_2_O_4_/NiCo_2_O_4_ compound system. At the same time, the morphological transformation of the NiMn_2_O_4_ sample to the Co-substituted samples take place in the significant conversion from irregular particles to well-distributed hollow multi-porous mesocrystals. The optimized NiCo_1.5_Mn_0.5_O_4_ sample maintains 532.2 mAh g^−1^ with 90.4% capacity retention after 100 cycles at a current density of 1 A g^−1^ with the voltage of 0.01–3.00 V vs. Li/Li^+^ at room temperature, because the superlattice NiMn_2_O_4_/NiCo_2_O_4_ structure can enhance the charge transport kinetics, and the uniform hollow multi-porous architecture improve the structural stability and facilitate the electrolyte penetration during lithiation and delithiation cycling.

## Experimental

### Materials synthesis

NiMn_2−x_Co_*x*_O_4_ (*x* = 0, 0.5, 1, 1.5, 2) oxide materials are synthesized by solvothermal reaction. The stoichiometric ratio of Ni(CH_3_COO)_2_·4H_2_O, Mn(CH_3_COO)_2_·2H_2_O, Co(CH_3_COO)_2_·2H_2_O, and hexamethylenetetramine (HMT) are dissolved in a mixed solvent of 13 mL water and 67 mL triethylene glycol (TEG) to obtain a homogeneous and transparent solution. Then the solution is put into a Teflon-lined stainless steel autoclave. After the autoclave is maintained at 200°C for 16 h, it is cooled to room temperature naturally. The precipitate is collected after filtration, washing, and drying to obtain the precursor. The as-prepared precursor is first calcinated at 250°C in air for 3 h and then at 900°C in air for 3 h.

NiMn_2−x_Co_*x*_O_4_ (*x* = 0, 0.5, 1, 1.5, 2) oxide materials, are labeled as NM (*x* = 0), Co-1 (*x* = 0.5), Co-2 (*x* = 1), Co-3 (*x* = 1.5), and Co-4 (*x* = 2), respectively, and these corresponding precursors are labeled as NMP, CoP-1, CoP-2, CoP-3, and CoP-4, respectively.

### Materials characterizations

X-ray diffraction (XRD, Rigaku D/Max 200PC, Japan) is employed to characterize the phases of all samples. The scanning range of diffraction angle (2θ) is 10°~90° and the scanning rate is 5° min^−1^. The morphologies of all samples are examined by scanning electron microscopy (SEM, Nova NanoSEM-230). The phase structures of the NM and Co-3 are analyzed by high resolution transmission electron microscopy (HRTEM, FEI Tecnai G2 F20 S-Twin working at 200 kV). The energy dispersive X-ray spectroscopy (EDS) is used for the elemental characterization of all samples by OXFORD 7426 as the attachment of SEM, with the acceleration voltage of 20 kV. A specific surface area (SSA) analysis was used to measure material's BET SSA (SSAA, 3H2000, BSD, China).

### Electrochemical measurement

The electrochemical tests are performed in CR2025 coin-type cells. Typical working electrode are prepared by a slurry coating process and the loadings are between 1.1 and 1.2 mg cm^−2^, and an electrode diameter of 10 mm is used. The working electrode consists of as-synthesized anode material, acetylene black and polyvinylidene difluoride (PVDF) binder with a weight ratio of 5:3:2. A lithium metal foil is used as the counter electrode. 1 M LiPF_6_ dissolved in the mixtures of dimethyl carbonate (DMC), Ethyl Methyl Carbonate (EMC) and ethylene carbonate (EC) with a volume ratio of 1:1:1 is used as electrolyte. The assembly of the cells is carried out in a dry an argon-filled MIKROUNA Universal 24401750 glovebox. Electrochemical performance of the materials are tested using an automatic galvanostatic charge-discharge unit, Land CT2001 battery cycler, in a cutoff voltage range within 0.01–3.0 V vs. Li/Li^+^ at room temperature. The cyclic voltammetry (CV) are operated at a scan rate of 0.1 mV s^−1^ in a cutoff voltage range within 0.01–3.0 V at room temperature with a CHI660D electrochemical analyzer. Finally, the electrochemical impedance spectroscopy (EIS) measurements are conducted by a CHI660D impedance analyzer, using 2-electrode cells. All cells are initially discharged and charged at a current density of 0.05 A g^−1^, and then discharged and charged different times of 1st and 100th at a current density of 1 A g^−1^ with the voltage of 0.01 – 3.00 V vs. Li/Li^+^ at room temperature. The amplitude voltage is 5 mV and the frequency range is 0.01–100,000 Hz.

## Results and discussion

**Figure 1:**
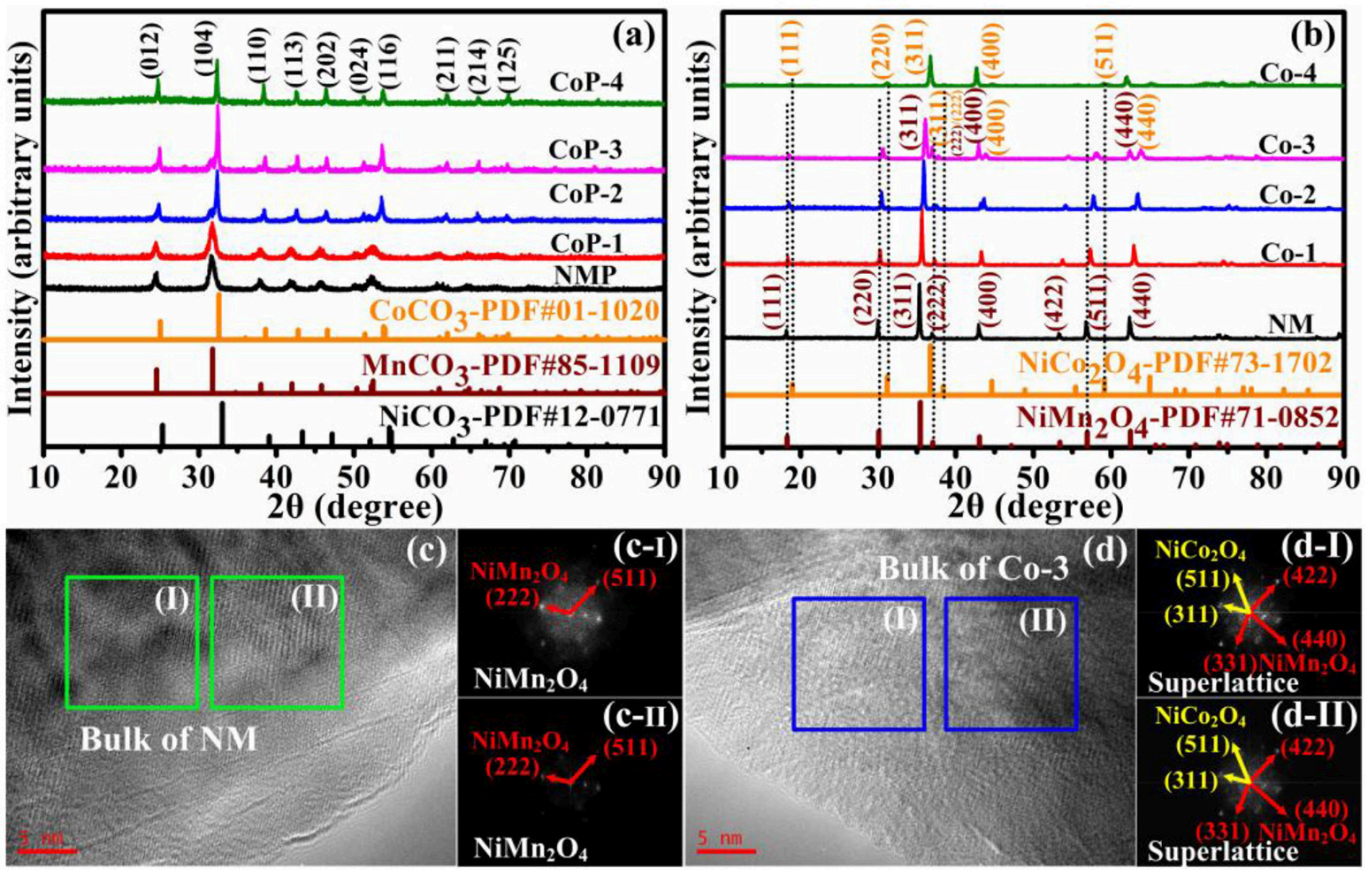
**(a)** X-ray diffraction patterns of NMP, CoP-1, CoP-2, CoP-3, and CoP-4, **(b)** X-ray diffraction patterns of NM, Co-1, Co-2, Co-3, and Co-4, HRTEM images of **(c)** NM and **(d)** Co-3 powders, and corresponding FFT (Fast Fourier transform) images.

Figure 1a displays the X-ray diffraction patterns of the NMP, CoP-1, CoP-2, CoP-3, and CoP-4 precursors. Compared with standard XRD patterns of NiCO_3_ (JCPDS no. 12-0771), MnCO_3_ (JCPDS no. 85-1109), and CoCO_3_ (JCPDS no. 01-1020), all the diffraction lines could be indexed on the basis of the hexagonal phase with the space group of R-3c (Qiu et al., 2011; Sun et al., 2014; Choi et al., 2015; Kang et al., 2015; Wu F. X. et al., 2016). With the increase of cobalt concentration, the manganese concentration decreases, and the diffraction peaks shift right to the positions at large angles, indicating that the substituted Co incorporates into the crystal lattice, resulting in the decrease of the lattice constant. Furthermore, from the diffraction lines of CoP-2 and CoP-3 precursors, it is noticed that a second phase appears in the pattern of the carbonate precursors at 2θ of about 32° and become obviously with the increase of the Co concentration, which can confirm that high cobalt concentrations prefer to form two phases rather than one phase (Ni_1/3_Mn_2/3−x_Co_*x*_CO_3_) in the carbonate precursors through the solvothermal process.

Figure 1b shows the X-ray diffraction patterns of the NM, Co-1, Co-2, Co-3, and Co-4 samples. The diffraction peaks of the NM sample in the XRD patterns can be assigned to well-crystallized cubic spinel NiMn_2_O_4_ (JCPDS no. 71-0852). Compared to that of the NM, the diffraction peaks of the Co-1 and Co-2 shift right to the positions at high difraction angles, as the cobalt concentration increases, because the lattice constant is decreased by substituting Co^3+^ (0.068 nm) into Mn^3+^ (0.072 nm) site (Suo et al., 2013; Mo et al., 2015; He et al., 2017). Furthermore, from the diffraction line of Co-3, the distinct peaks of (311), (400), and (440) at 2θ of about 36, 43, and 64° are all completely separated, respectively, indicating a second cubic spinel phase of NiCo_2_O_4_, which is in accordance with the precursors results. These results suggest that the Co-3 is consistently made up of two phases, which allows hybridizing the phase of NiMn_2_O_4_ with the phase of NiCo_2_O_4_ into the architecture. In addition, the diffraction lines of Co-4 is assigned to CoO (JCPDS no. 70-2855) and NiCo_2_O_4_ (JCPDS no. 73-1702), which might be ascribed that the single phase NiCo_2_O_4_ cannot be synthesized through our heat-treat process. Chen et al. (Mo et al., 2015) reported that NiCo_2_O_4_ is synthesized at 400°C with a temperature ramp of 4°C min^−1^ and kept for 5 h under ambient air. However, in our study, Co-4 was first calcinated at 250°C in air for 3 h and then at 900°C in air for 3 h, which is consistency with the other samples.

To further confirm the effect of Co substitution on the microstructure of NiMn_2_O_4_, HRTEM and corresponding FFT (fast Fourier transform) images of NM and Co-3 samples are exhibited in Figures 1c,d. The lattice fringes and FFT patterns of both samples reveal that the NiMn_2_O_4_ structure is successfully formed as expected. However, compared to that of NM (c-I and c-II), the corresponding FFTs of Co-3 (d-I and d-II) exhibit another two interplanar distances of 1.56 and 2.45 Å, which corresponds to the d-spacing of the (511) and (311) planes of NiCo_2_O_4_ (JCPDS no. 73-1702), respectively. The NiMn_2_O_4_ and NiCo_2_O_4_ phases are mixing with one another at the atomic level in Co-3 particle, which suggests a two-phase composite nature of the sample derived from small fields of view. These results can determine that the Co-3 is a new mixed-phase NiMn_2_O_4_/NiCo_2_O_4_ compound system with the presence of a superstructure. In addition, it is reported that the chemical composition of Ni and Co elements in NiCo_2_O_4_ contains Ni^2+^/Ni^3+^ and Co^2+^/Co^3+^, and the chemical composition of Ni and Mn elements in NiMn_2_O_4_ contains Ni^2+^/Ni^3+^ and Mn^2+^/Mn^3+^ (Kang et al., 2015; Ma Y. et al., 2015; Mo et al., 2015; Shen et al., 2015; Li et al., 2016). Therefore, we hold this assumption that the chemical composition of Ni Co and Mn elements in NiCo_1.5_Mn_0.5_O_4_ contains Ni^2+^/Ni^3+^, Co^2+^/Co^3+^, and Mn^2+^/Mn^3+^.

**Figure 2:**
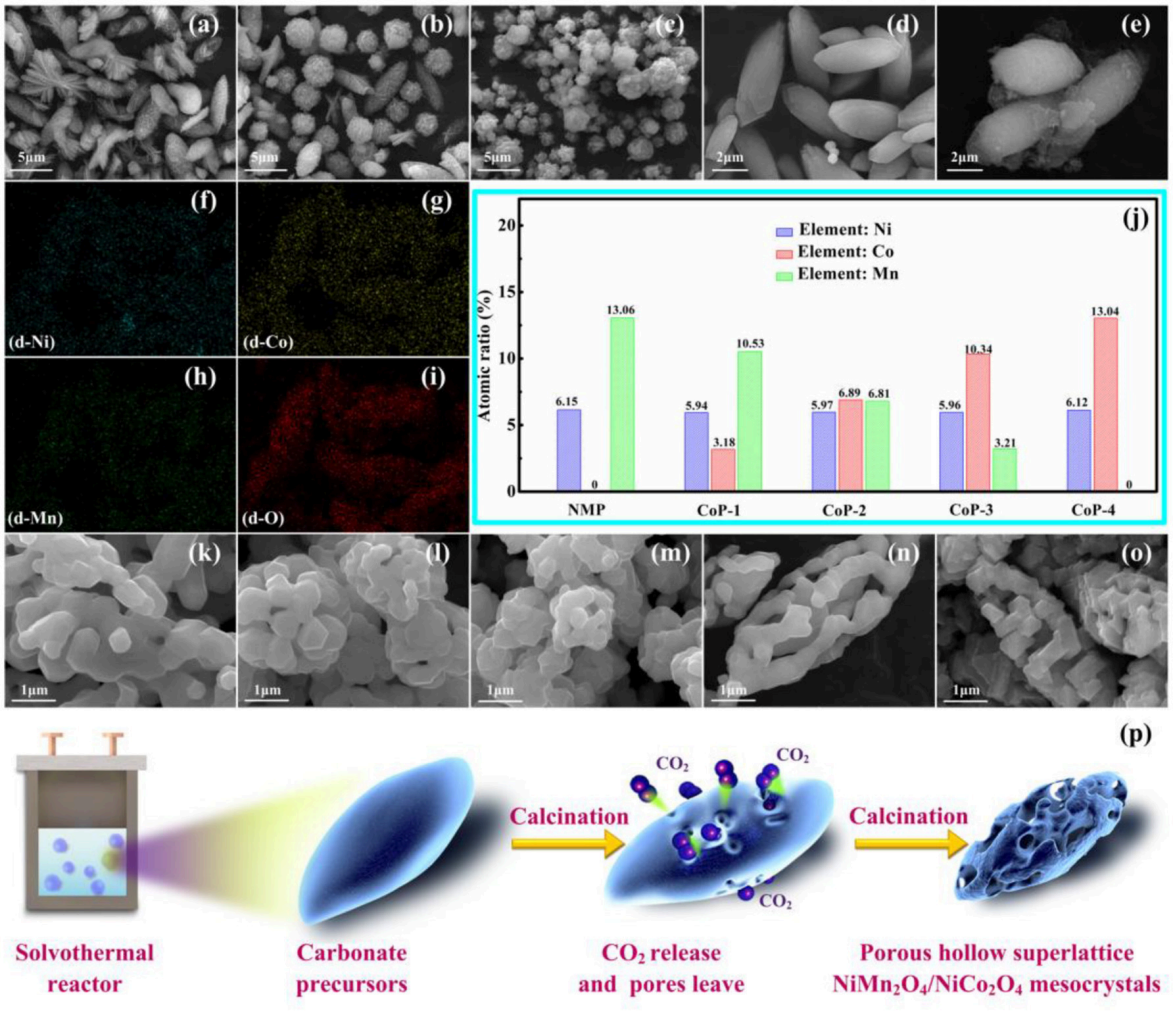
SEM images of **(a)** NMP, **(b)** CoP-1, **(c)** CoP-2, **(d)** CoP-3, and **(e)** CoP-4, corresponding EDS mappings of Ni **(f)**, Co **(g)**, Mn **(h)**, and O **(i)** for **(d)** CoP-3, and the EDX contents **(j)** of Ni, Co and Mn element in NMP, CoP-1, CoP-2, CoP-3, and CoP-4, SEM images of **(k)** NM, **(l)** Co-1, **(m)** Co-2, **(n)** Co-3, and **(o)** Co-4, Schematic diagram **(p)** for the synthesis process of the porous hollow superlattice NiMn_2_O_4_/NiCo_2_O_4_ mesocrystals.

The SEM images of all carbonate precursors, corresponding EDS mappings of Ni, Co, Mn, and O for CoP-3 carbonate precursors and the EDX contents of Ni, Co and Mn element in all carbonate precursors are illustrated in Figures 2a–j. As can be seen in Figures 2a,b, the NMP and CoP-1 are composed by microflakes, microspheres and spindle-like particles. With the increase of Co concentration (Figures 2c–e), the microflakes disappear, and the microspheres are transformed into well-distributed spindle-like particles. EDS mapping result confirms that all elements, including Co, are uniformly distributed. EDX analysis exhibits that the ratio of Ni, Co and Mn element in NMP, CoP-1, CoP-2, CoP-3, and CoP-4 is about 1:0:2, 1:0.5:1.5, 1:1:1, 1:1.5:0.5, 1:2:0, respectively, which is in accordance with our expectation. Therefore, it could be concluded that the stoichiometrical precursors are successfully synthesized via hydrothermal process, and that Co substitution is conducive to the good morphological consistency and distribution of the NMP particles.

The SEM images of all samples are illustrated in Figures 2k–o and Figure S1. Particles possessing porous character are observed for all the samples, which could be ascribed to the decomposition of carbonate precursor during heating process. It is also noted that the particles morphology for all the samples are inherited from those of the precursors. For example, particles from the NM sample show irregular morphology and random distribution. However, for the Co-1 and Co-2, the particles exhibit hollow spherical morphology and good distribution. As the increase of Co concentration, particles from the Co-3 exhibit spindle-like morphology and hollow multi-porous architecture. EDS mapping of Co-3 (Figure S1) shows a uniform distribution of Ni, Co, Mn, and O throughout the particle. These results confirm that Co incorporates into the Co-3, and Co substitution changes the morphology of both primary and aggregate particles, which might be benefit for lithium and electron diffusion during lithiation/delithiation reaction.

Schematic diagram for the synthesis process of the porous hollow superlattice NiMn_2_O_4_/NiCo_2_O_4_ mesocrystals are illustrated in Figure 2p. The acetate molecules and hexamethylenetetramine dissolve in the solvent then gather together to form small droplets. During solvothermal treatment, the hexamethylenetetramine decomposes, and creates CO_2_ bubbles. By capturing the metal ions on bubble surface, an oval-like microsphere precursor is obtained successfully. During calcination process, metal ions move into crystal structure of the superlattice NiMn_2_O_4_/NiCo_2_O_4_ mesocrystals, meanwhile, CO_2_ release in the carbonate precursors, leaving pores on the obtained hollow complex oxides (Luo D. et al., 2014; Luo et al., 2015; Ma Z. et al., 2015; Shen et al., 2015).

**Figure 3:**
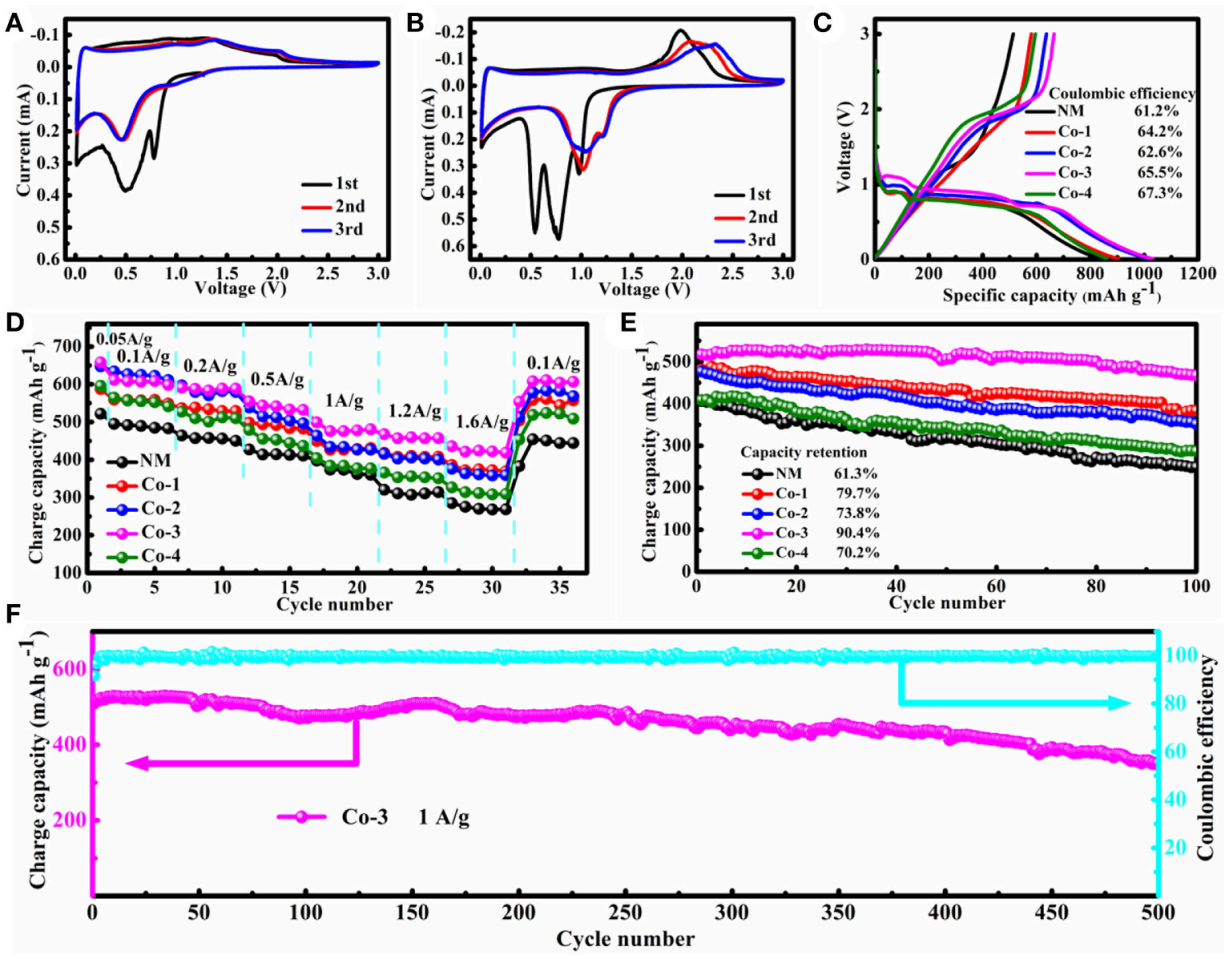
Electrochemical performance: **(A)** CV plots of NM, **(B)** CV plots of Co-3, **(C)** initial charge-discharge curves of NM, Co-1, Co-2, Co-3 and Co-4 (0.05 A g^−1^), **(D)** rate capability (0.05 A g^−1^-1.6 A g^−1^), **(E)** cyclic ability (1 A g^−1^) of all samples, **(F)** long-term cyclic ability of Co-3 (1 A g^−1^).

To understand the oxidation/reduction and phase transformation processes in electrode reactions of the synthesized samples, the CV curves for the initial three cycles of NM and Co-3 samples are presented in Figures 3A,B, which are tested at a scan rate of 0.1 mV s^−1^ in the voltage range of 0.01–3.00 V vs. Li/Li^+^ at room temperature, respectively. The CV curves of both samples for the first cycle are significantly different from those for the following cycles, because of the irreversible electrochemical reaction during the first discharge cycle. However, no obvious alteration is observed between the second cycle and the third cycle, suggesting the good reversibility of lithium insertion and extraction reactions. In the first cycle, as can be seen in Figure 3A, there is a sharp reduction peak at 0.9 V and a broad reduction peak between 0.3 and 0.8 V. The sharp one associates with the initial reduction of Mn^3+^–Mn^2+^, and the broad one can be attributed to the reduction of NiMn_2_O_4_ to metallic Mn and Ni and the formation of Li_2_O (Li J. F. et al., 2013b; Kang et al., 2015; Ma Y. et al., 2015; Ma Z. et al., 2015). With the increase of Co concentration (Figure S2), it is clear that the sharp reduction peak at 0.9 V is disappeared, and the reduction peaks which is attributed to the reduction of the oxides to metal and the formation of Li_2_O are narrower than that of NiMn_2_O_4_. In comparison, as shown in Figure 3B, a new reduction peak appear at 1.0 V, which can be corresponded to the reduction of Co^3+^–Co^2+^, and the broad reduction peaks of Co-3 are completely separated. Yuan et al. (Ma Z. et al., 2015) and Lee et al. (Kang et al., 2015) reported that “the sharp peak at 0.5 V should correspond to the reduction of NiMn_2_O_4_ to metallic Mn and Ni,” while Yang et al. (Zhang et al., 2016) and Wang et al. (Leng et al., 2015) reported that “the peak at 0.8 V should correspond to the reduction of NiCo_2_O_4_ to metallic Co and Ni,” and both peaks “relate to the formation of Li_2_O and solid-electrolyte interface (SEI)” (Li J. F. et al., 2013c; Zhang et al., 2014, 2018; Gao et al., 2015). These results further confirm that Co-3 is a kind of mixed-phase NiMn_2_O_4_/NiCo_2_O_4_ composite material, which is in accordance with the XRD results and TEM results as aforesaid. For the Co-3 sample, in the anodic sweep, a wide oxidation peak (~2.0 V) can be attributed to the oxidation of metals to oxides accompanying the decomposition of Li_2_O. Based on the above CV analysis, in conjunction with the previously reported storage mechanism for NiMn_2_O_4_ and NiCo_2_O_4_ (Li J. F. et al., 2013c; Zhang et al., 2014, 2016, 2018; Gao et al., 2015; Kang et al., 2015; Leng et al., 2015; Ma Y. et al., 2015; Ma Z. et al., 2015; Su et al., 2015), the electrochemical reactions for the electrodes can be summarized as follows:

(1) $$\begin{array}{r} \text{NiM}{\text{n}}_{2}{\text{O}}_{4}\;+\;8\text{L}{\text{i}}^{+}\;+\;8{\text{e}}^{-}\;\to \text{ Ni }+\;2\text{Mn }+\;4\text{L}{\text{i}}_{2}\text{O} \end{array}$$

(2) $$\begin{array}{r} \text{Ni }+\;2\text{Mn }+\;3\text{L}{\text{i}}_{2}\text{O }↔\text{ NiO }+\;2\text{MnO }+\;6\text{L}{\text{i}}^{+}+\;6{\text{e}}^{-} \end{array}$$

(3) $$\begin{array}{r} \text{NiC}{\text{o}}_{2}{\text{O}}_{4}\;+\;8\text{L}{\text{i}}^{+}\;+\;8{\text{e}}^{-}\;\to \text{ Ni }+\;2\text{Co }+\;4\text{L}{\text{i}}_{2}\text{O} \end{array}$$

(4) $$\begin{array}{r} \text{Ni }+\;2\text{Co }+\;3\text{L}{\text{i}}_{2}\text{O }↔\text{ NiO }+\;2\text{CoO }+\;6\text{L}{\text{i}}^{+}+\;6{\text{e}}^{-} \end{array}$$

(5) $$\begin{array}{r} \text{NiM}{\text{n}}_{2-\text{x}}\text{C}{\text{o}}_{\text{x}}{\text{O}}_{4}\;+\;8\text{L}{\text{i}}^{+}\;+\;8{\text{e}}^{-}\;\to \text{ Ni }\\ \;+\;\left(2-x\right)\text{Mn}+\;x\text{Co }+\;4\text{L}{\text{i}}_{2}\text{O} \end{array}$$

(6) $$\begin{array}{rc} \text{Ni }+\;\left(2-x\right)\text{Mn }+\;x\text{Co }+\;3\text{L}{\text{i}}_{2}\text{O }↔\text{ NiO }\\ \; & \;+\;\left(2-x\right)\text{MnO}+\;x\text{CoO }+\;6\text{L}{\text{i}}^{+}\;+\;6{\text{e}}^{-} \end{array}$$

Figure 3C depicts the initial charge-discharge curves of the NM, Co-1, Co-2, Co-3, and Co-4 samples. The electrochemical tests are carried out at a current density of 0.05 A g^−1^ with the voltage of 0.01–3.00 V vs. Li/Li^+^ at room temperature. As can be seen in Figure 3C, there is a representative plateaus of the profile for NM around 0.7 V in the first discharge process. By comparison, a representative profile of Co-3 which is composed of two plateaus around 0.9 V and 0.7 V in the first discharge process can be observed clearly, which relates to the insertion of lithium into the crystal structure of NiCo_2_O_4_ and NiMn_2_O_4_, respectively, accompanied by the formation of Li_2_O (Wang J. X. et al., 2014; He et al., 2015, 2017; Mo et al., 2015; Li et al., 2016). The initial charge capacity for the NM, Co-1, Co-2, Co-3, and Co-4 is 513.5, 580.8, 636.9, 665.4, and 595.7 mAh g^−1^, respectively. It is obvious that the initial charge capacities of the Co-substituted samples are superior than that of the NM, particularly, the Co-3 sample exhibit the optimal initial charge capacities of all samples.

The rate capability of all samples are given in Figure 3D. The cells are charged/discharged at a current density of 0.05 A−1.6 A g^−1^, and then charged/discharged at a current density of 0.1 A g^−1^ with the voltage of 0.01–3.00 V vs. Li/Li^+^ at room temperature. It is noted that the Co-substituted samples show improved rate capability compared to the pristine counterparts at high rates. Specifically, the Co-3 demonstrates the best rate performance and delivers the highest reversible capacities of all samples. Remarkably, it can maintain the charge capacities of 611.4, 591.1, 554.6, 499.1, 467.6, 435.6, and 606.8 mAh g^−1^ after 5 cycles at the current density of 0.1, 0.2, 0.5, 1, 1.2, 1.6, and 0.1 A g^−1^, respectively. Even at a current density as high as 1.6 A g^−1^, the Co-3 can still deliver a stable capacity of about 435.6 mAh g^−1^ after 5 cycles, which is still higher than the theoretical capacity of the graphite (372 mAh g^−1^), indicating the excellent rate capability.

The comparison of the cycling stability between the NM and the Co-substituted samples are presented in Figure 3E. The cells were charged/discharged 2 times at a current density of 0.05 A g^−1^, and then charged/discharged 100 times at a current density of 1 A g^−1^ with the voltage of 0.01–3.00 V vs. Li/Li^+^ at room temperature. The charge capacity retention reaches 61.3, 79.7, 73.8, 90.4, and 70.2% after 100 cycles for NM, Co-1, Co-2, Co-3, and Co-4, respectively. In particular, as can be seen in Figure 3F, the Co-3 maintains 532.2 mAh g^−1^ with 67.8% capacity retention after 500 cycles at a current density of 1 A g^−1^ with the voltage of 0.01–3.00 V vs. Li/Li^+^ at room temperature. It means that the Co-3 can still deliver a stable capacity of about 360.8 mAh g^−1^ after 500 cycles, which is still near the theoretical capacity of the graphite (372 mAh g^−1^), demonstrating the outstanding cycling stability. In conclusion, the porous Co-substituted samples in this work exhibit a superior electrochemical performance compared with the NM, specially, the Co-3 exhibits the optimal electrochemical performance of all samples.

**Figure 4:**
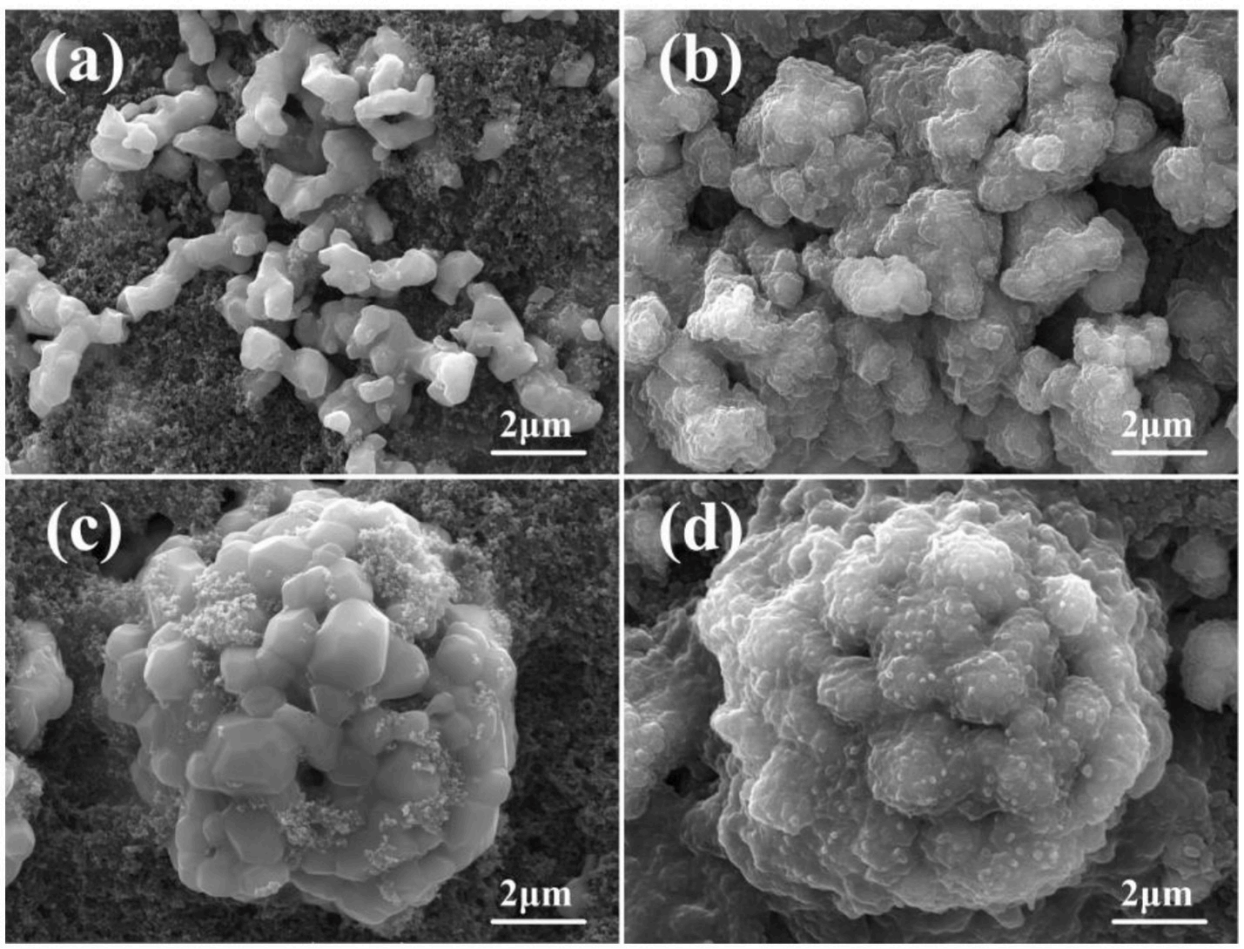
SEM images of **(a,b)** NM electrodes, **(c,d)** Co-1 electrodes, **(a,c)** before cycling, **(b,d)** after 100 cycles at a current density of 1A g^−1^ with the voltage of 0.01–3.00 V vs. Li/Li^+^.

**Figure 5:**
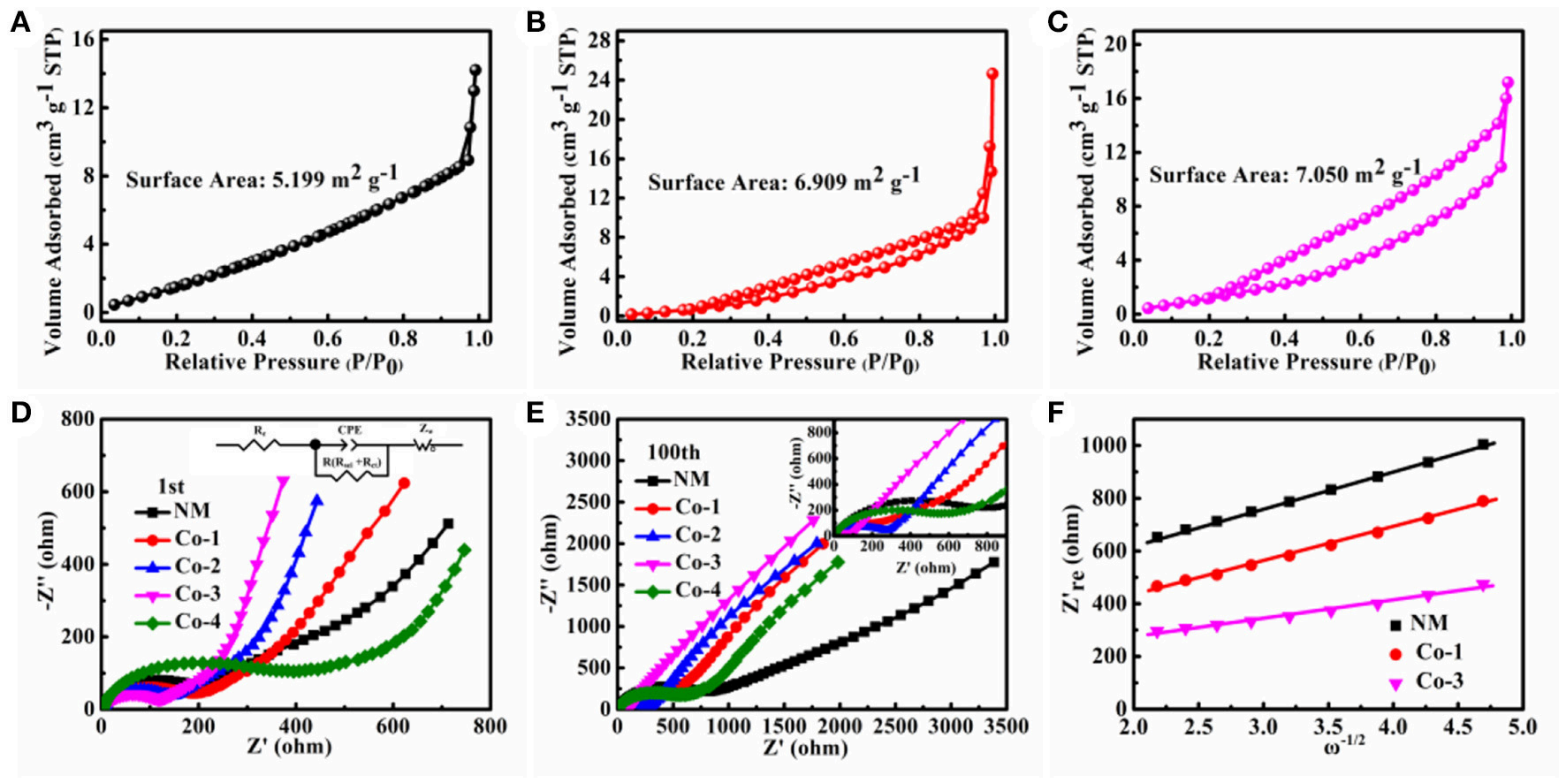
N_2_ adsorption and desorption isotherm: **(A)** NM, **(B)** Co-1, and **(C)** Co-3. Nyquist plots of as-prepared electrodes, **(D)** after the 1st cycle and **(E)** after the 100th cycle, **(F)** the relationships between Z'_re_ and ω^−1/2^ after the 1st cycle for the NM, Co-1 and Co-3 electrodes.

To further understand the effects of the porous architecture on the electrochemical properties of Co-substituted samples, the Co-1 is selected as a reference, which has the similar structure but different morphological architecture, compared to the NM. The SEM images of NM and Co-1 electrodes before and after 100 cycles are shown in Figure 4. The cells are charged to 3.00 V at high delithiation state. As can be seen in Figure 4, after 100 cycles, both samples (Figures 4b,d) show fuzzy surface, which may be due to the formation of Li_2_O and the electrolyte decomposition (Wang J. X. et al., 2014; Mo et al., 2015; Li et al., 2016; He et al., 2017). Compared to the fresh electrode (Figure 4a), the irregular morphological NiMn_2_O_4_ particles suffer serious damage and expand into aggregate particles after 100 cycles (Figure 4b). The aggregation can be ascribed to the volume expansion on account of the irreversible electrochemical process from the high lithiation state (Pan et al., 2014; Zhang et al., 2015). In contrast, for Co-1, as shown in Figures 4c,d, there is no obvious alteration in the hollow multi-porous particle morphology after 100 cycles. These results demonstrate that the hollow multi-porous architecture can provide enough void spaces to alleviate the architectural change, and offer a stable structure for the intercalation and de-intercalation cycling (Zhang et al., 2015). In addition, Nitrogen adsorption/desorption isotherms are shown in Figures 5A–C, the sorption isotherms are of type III isotherms, they have a distinct H3-type hysteresis loop in the range of P/P_0_ = 0.2–0.99, which does not close until the saturation pressure is reached. Obviously, the BET specific surface area (SSA) of the Co-1 is 6.909 m^2^ g^−1^, which is larger than that of the NM sample (5.199 m^2^ g^−1^). The larger SSA can be derived from the hollow multi-porous architecture, leading to a shorter Li ions diffusion distance during the charge-discharge processes.

Furthermore, nyquist plots of the as-prepared electrodes and corresponding EIS and lithium ion diffusion coefficient (D_Li+_) results are exhibited in Tables 1, 2 and Figures 5D–F. The semicircle at high and intermediate frequency region relates the SEI film impedance (R_*sei*_) and the charge transfer impedance (R_*ct*_), respectively. The inclined straight line at the low frequency region is attributed to Warburg impedance (R_*w*_), which is associated with Li^+^ diffusion into the bulk (Qiu et al., 2011; Li et al., 2012; Chen et al., 2014a,b; Sun et al., 2014; Wang L. Y. et al., 2014; Xu et al., 2014; Choi et al., 2015; Wu F. X. et al., 2016; Wu et al., 2018). The lithium ion diffusion coefficient was calculated via a widely accepted method (Reddy et al., 2013; Li et al., 2015). With the increase of cycle number, it is obvious that the values of R for all samples rise. After 100 cycles, the resistance of all samples increase, and both samples (Figures 4b,d) show fuzzy surface. These results indicate that the SEI formed on the electrode. In addition, as shown in Figures 4c,d, there is no obvious alteration in the hollow multi-porous particle morphology after 100 cycles, which suggests that the uniform hollow multi-porous architecture can alleviate the pore size/function change originating from the volume expansion and the SEI formation during cycling. Specially, the value of R for NM after 1 cycle and 100 cycles is 218.5 and 719.3 Ω, respectively, the dramatical increase of the resistance could be ascribed to the formation and expansion of aggregate particles (as seen in Figure 4), which is certainly harmful to electrolyte penetration and the lithium ions and electron transport. In comparison, the Co-substituted samples do not have significant changes, which should be attributed to that the hollow multi-porous architecture can provide enough void spaces to prevent particle aggregation, resulting in enhanced the charge transport kinetics during discharging/charging processes (Zhang et al., 2015). It is clear from the above discussions that the excellent rate capability and cycle stability of Co-substituted samples are attributed to the porous architecture by following factors: (1) provides enough void spaces to suppress the architectural collapse during cycle, (2) reduces the lithium ions diffusion and electron-transportation distances; (3) facilitates the electrolyte penetration resulting in enhanced the charge transport kinetics.

**Table 1:** The values of R for NM, Co-1, Co-2, Co-3 and Co-4 samples after the 1st and 100th cycle.

**Samples**	**NM**	**Co-1**	**Co-2**	**Co-3**	**Co-4**
1st (Ω)	218.5	187.3	136.1	115.7	372.4
100th (Ω)	791.3	277.1	281.5	127.6	568.2

**Table 2:** The values of specific surface area and D_Li+_ for the NM and Co-3 samples.

**Samples**	**NM**	**Co-1**	**Co-3**
D_Li+_(cm^2^ S^−1^)	2.99 × 10^−12^	3.49 × 10^−12^	1.19 × 10^−11^
BET SSA (m^2^ g^−1^)	5.199	6.909	7.050

To further evaluate the effects of the superlattice structure on electrochemical properties of the NiMn_2_O_4_/NiCo_2_O_4_, the electrochemical kinetics of the Co-3 and Co-1 samples have been studied. The reason why choose Co-3 and Co-1 is partly due to the Co-3 exhibits superior electrochemical properties and superlattice structure, and partly due to Co-1 owns similar porous morphology with the Co-3, while is made up with a single phase. The BET results reveal that the SSA of the Co-1 and Co-3 samples is 6.909 and 7.050 m^2^ g^−1^, respectively. The similar SSA means the similar Li ions diffusion distance during the charge-discharge process. Therefore, it is supposed that the electrochemical kinetic difference between the Co-1 and Co-3 mainly lies on structure. As shown in Table 1, the Co-3 exhibits the lowest initial R and the most stable resistance during cycling among all samples. Furthermore, it can be seen from Table 2, the D_Li_+ of the Co-3 sample is 1.19 × 10^−11^ cm^2^ S^−1^, which is much higher than that of the Co-1 sample (3.49 × 10^−12^ cm^2^ S^−1^). Since the morphology and the SSA of the Co-1 is similar to those of Co-3, the superior electron and Li ion transport diffusive capacity of the Co-3 should be related to the superlattice NiMn_2_O_4_/NiCo_2_O_4_ structure. A similar phenomenon has also been reported by Chen et al (Suo et al., 2013). They pointed out that the composite phases can supply high ionic conductivity. These results demonstrate that the superlattice NiMn_2_O_4_/NiCo_2_O_4_ structure can facilitate the ionic conductivity to enhance charge transport kinetics of the bulk material.

**Scheme 1:**
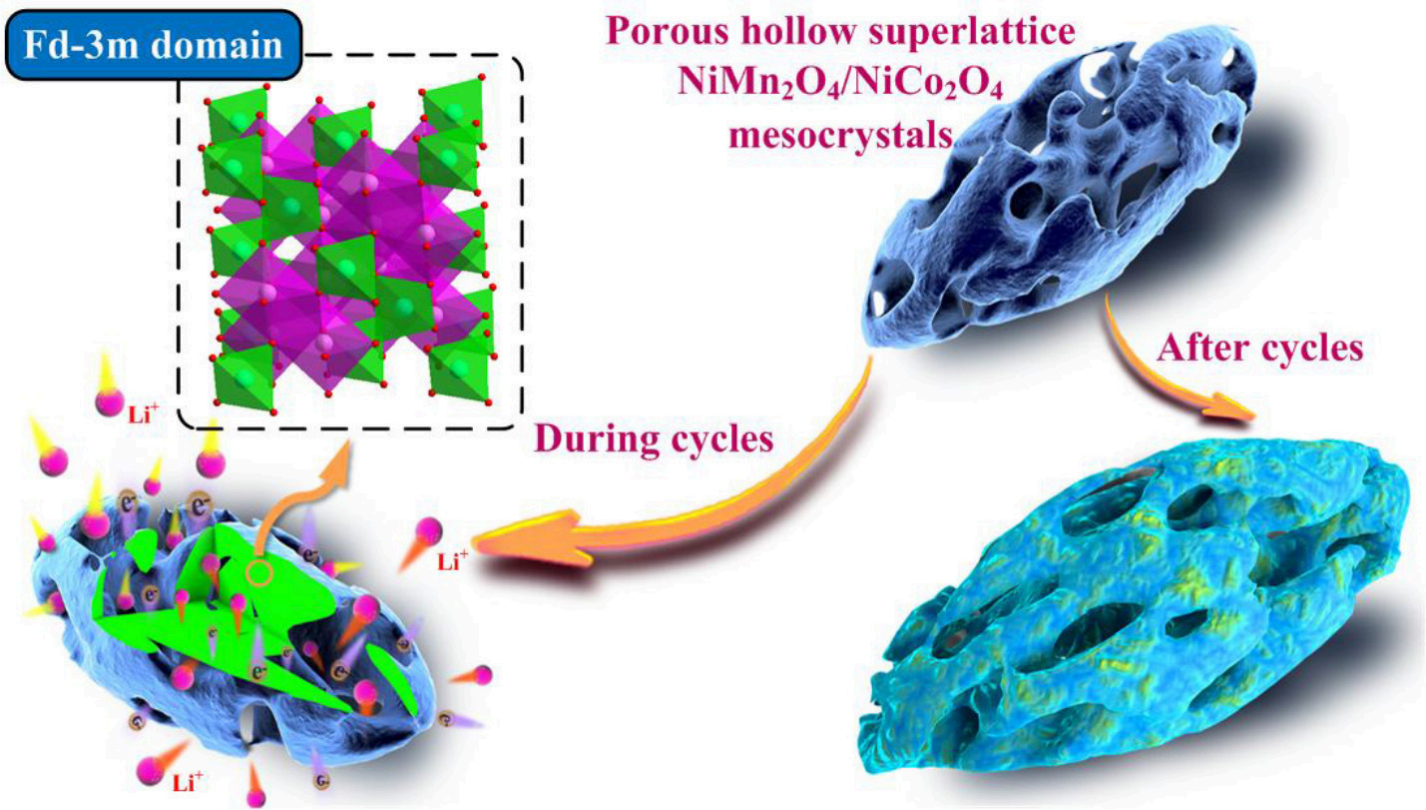
Schematic illustrations of the porous hollow superlattice NiMn_2_O_4_/NiCo_2_O_4_ mesocrystals and its function during cycles and after cycles.

The schematic illustrations of the porous hollow superlattice NiMn_2_O_4_/NiCo_2_O_4_ compound and the mechanism of electrochemical process are exhibited in Scheme 1. Many reports (Kang et al., 2015; Ma Y. et al., 2015; Ma Z. et al., 2015; Zhang et al., 2015, 2016) indicate that the aggregation from the irreversible volume expansion and the low charge transport kinetics, including the poor intrinsic electronic conductivity and the slow ion-diffusion rates, is responsible for the fading capacity of NiMn_2_O_4_. In this work, the porous hollow architecture can provide enough void spaces to suppress architectural collapse originating from the volume expansion during cycling, and offer a short stable path for the lithium ions diffusion and electron-transportation. Moreover, the superlattice NiMn_2_O_4_/NiCo_2_O_4_ structure can facilitate the electronic and ionic conductivity to enhance charge transport kinetics of the bulk material. The synergistic effects of the superlattice NiMn_2_O_4_/NiCo_2_O_4_ structure and the uniform hollow multi-porous architecture are guaranteed for the excellent electrochemical performance.

## Conclusions

In summary, the Co substituted NiMn_2_O_4_ mesocrystals with controlled phase structure and morphology are successfully synthesized via a simple solvothermal method. The optimal sample with well-distributed hollow multi-porous superlattice NiMn_2_O_4_/NiCo_2_O_4_ mesocrystals exhibits excellent rate ability and enhanced cycling performance, which maintains 532.2 mAh g^−1^ with 90.4% capacity retention after 100 cycles, and can still deliver a stable capacity of about 360.8 mAh g^−1^ after 500 cycles, at a current density of 1 A g^−1^. The excellent electrochemical performance can be attributed to the synergistic effect of the superlattice NiMn_2_O_4_/NiCo_2_O_4_ structure and the uniform hollow multi-porous architecture. The superlattice structure can enhance the Li ion diffusion coefficient to enhance the charge transport kinetics. While, the uniform hollow multi-porous architecture can alleviate the architectural change originating from the volume expansion during cycling and facilitate electrolyte penetration. These combined effects make the NiCo_1.5_Mn_0.5_O_4_ a promising anode material for the practical application in EV/HEV and energy storage systems.

## Author contributions

LL and QY conceived the idea; QY and LL prepared all materials; QY, HY, and JL conducted SEM experiments; KY, BL, and ZL conducted XRD experiments; LL, QY, and BZ analyzed the data; QY wrote the manuscript and BZ, ZC, and JD commented on it; LL supervised the implementation of the project.

### Conflict of interest statement

The authors declare that the research was conducted in the absence of any commercial or financial relationships that could be construed as a potential conflict of interest. The reviewer, BQ, and handling Editor declared their shared affiliation.
