# Supplementary material for: Porous Hollow Superlattice NiMn2O4/NiCo2O4 Mesocrystals as a Highly Reversible Anode Material for Lithium-Ion Batteries
Source: Front Chem. 2018 May 15;6:153. doi: 10.3389/fchem.2018.00153 (PMC5962773; doi:10.3389/fchem.2018.00153)
Supplement: Supplementary file 1 [file Table_1.docx]

Porous Hollow Superlattice NiMn_2_O_4_/NiCo_2_O_4_ Mesocrystals as a Highly Reversible Anode Material for Lithium-Ion Batteries

*Qi Yao^a^, Lingjun Li^a, b ,^**^*^, Jiequn Liu^c^, Kaibo Ye^a^, Boyu Liu^a^, Zengsheng Liu^a^, Huiping Yang^a^, Zhaoyong Chen^a^, Junfei Duan^a^, Bao Zhang^d, *^*

AUTHOR ADDRESS

*^a^* School of Materials Science and Engineering, Changsha University of Science and Technology, Changsha 410114, P.R. China

*^b^* Hunan Provincial Key Laboratory of Efficient & Clean Energy Utilization, Changsha University of Science and Technology, Hunan Changsha 410004, P.R. China

*^c^* School of Iron and Steel, Soochow University, Suzhou 215021, P.R. China

*^d^* School of Metallurgy and Environment, Central South University, Changsha, 410083, P.R. China

CORRESPONDING AUTHOR

*^*^* Lingjun Li, Email: [lingjun.li@csust.edu.cn](mailto:csullj@hotmail.com)

*^*^* Bao Zhang, Email: [csuzb@vip.163.com](mailto:kaizhang@cityu.edu.hk)


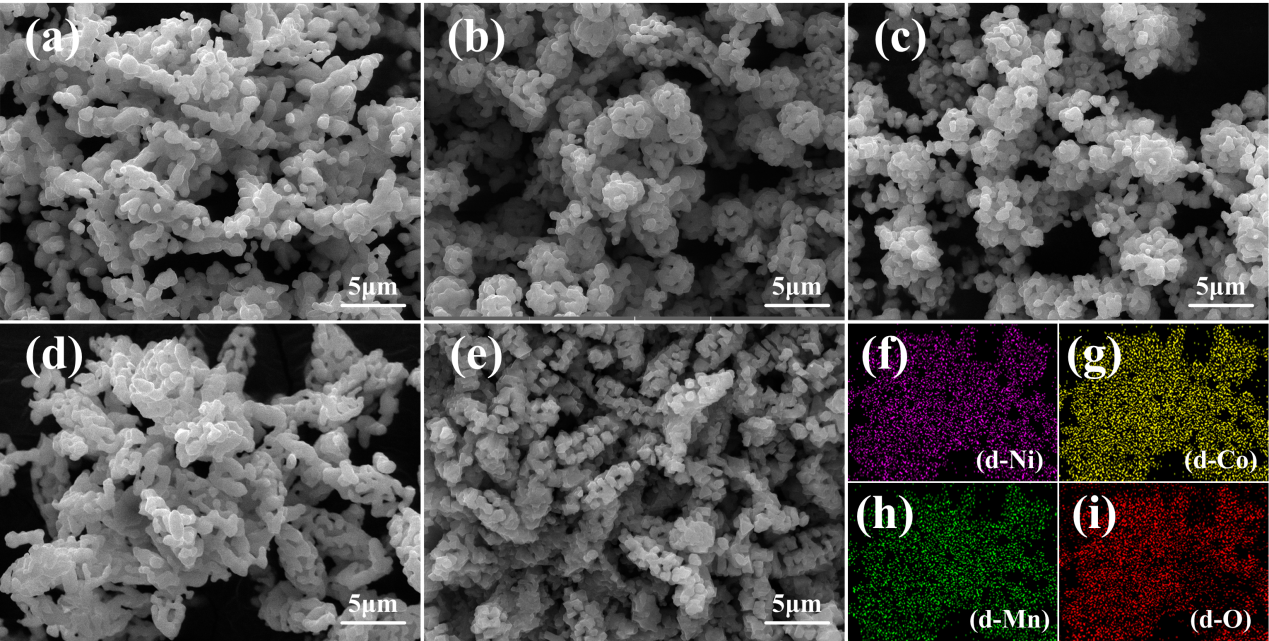


**Figure S1.** SEM images of (a) NM, (b) Co-1, (c) Co-2, (d) Co-3, and (e) Co-4, corresponding EDS mappings of Ni (f), Co (g), Mn (h) and O (i) for (d) Co-3


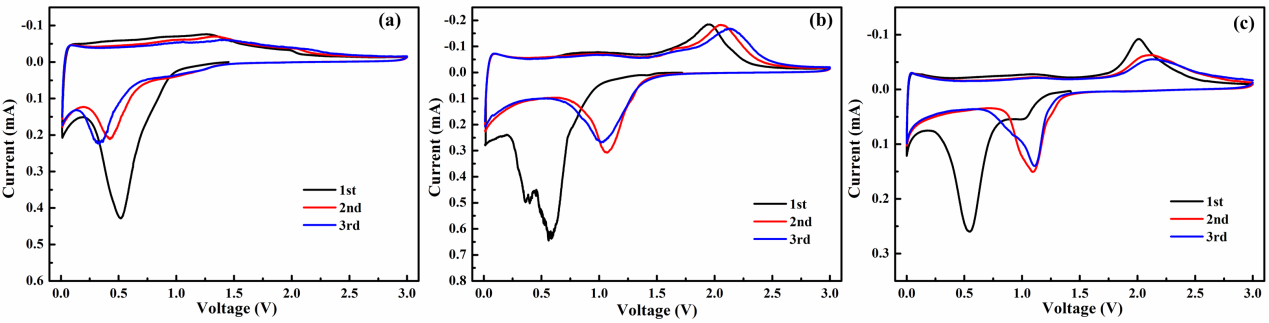


**Figure S2.** CV plots of (a) Co-1, (b) Co-2 and (c) Co-4

First discharge reaction: $\mathrm{Ni}\mathrm{Mn}_{2}O_{4}+8\mathrm{Li}^{+}+8e^{-} \leftrightarrow Ni+2Mn+4\mathrm{Li}_{2}O$ $\text{ NiMn}\text{2-}\text{x}\text{Co}\text{x}\text{O}\text{4}\text{ + 8Li}\text{+}\text{ + 8e}\text{-}\text{ → Ni + (2-}\text{x}\text{)Mn + }\text{x}\text{Co + 4Li}\text{2}\text{O}$

$$\text{NiMn}\text{2-}\text{x}\text{Co}\text{x}\text{O}\text{4}\text{ + 8Li}\text{+}\text{ + 8e}\text{-}\text{ → Ni + (2-}\text{x}\text{)Mn + }\text{x}\text{Co + 4Li}\text{2}\text{O}$$

NiCo_1.5_Mn_0.5_O_4_ Specific capacity=899 mA h g^-1^

Reversible reaction:

$\text{Ni + (2-}\text{x}\text{)Mn + }\text{x}\text{Co + 3Li}\text{2}\text{O ↔ NiO + (2-}\text{x}\text{)MnO + }\text{x}\text{CoO + 6Li}\text{+}\text{ + 6e}\text{-}$ $Ni+2Mn+3\mathrm{Li}_{2}O \leftrightarrow NiO+2MnO+6\mathrm{Li}^{+}+6e^{-}$

NiCo_1.5_Mn_0.5_O_4_ Specific capacity=674 mA h g^-1^
